# Extracellular Vesicles: Roles in Human Viral Infections, Immune-Diagnostic, and Therapeutic Applications

**DOI:** 10.3390/pathogens9121056

**Published:** 2020-12-17

**Authors:** Ayodeji O. Ipinmoroti, Qiana L. Matthews

**Affiliations:** 1 Microbiology Program, Alabama State University, Montgomery, AL 36104, USA; aipinmoroti0038@myasu.alasu.edu; 2 Department of Biological Sciences, College of Science, Technology, Engineering and Mathematics, Alabama State University, Montgomery, AL 36104, USA

**Keywords:** viral infection, extracellular vesicles, HIV, biophysical pathways, RNA, exosome, vesicles, antigen, membrane, markers

## Abstract

Membrane-bound vesicles that are released from cells are increasingly being studied as a medium of intercellular communication, as these act to shuttle functional proteins, such as lipids, DNA, rRNA, and miRNA, between cells during essential physiological processes. Extracellular vesicles (EVs), most commonly exosomes, are consistently produced by virus-infected cells, and they play crucial roles in mediating communication between infected and uninfected cells. Notably, pathophysiological roles for EVs have been established in various viral infections, including human immune deficiency virus (HIV), coronavirus (CoV), and human adenovirus (HAdv). Retroviruses, such as HIV, modulate the production and composition of EVs, and critically, these viruses can exploit EV formation, secretion, and release pathways to promote infection, transmission, and intercellular spread. Consequently, EV production has been investigated as a potential tool for the development of improved viral infection diagnostics and therapeutics. This review will summarize our present knowledge of EV–virus relationships, focusing on their known roles in pathophysiological pathways, immunomodulatory mechanisms, and utility for biomarker discovery. This review will also discuss the potential for EVs to be exploited as diagnostic and treatment tools for viral infection.

## 1. Introduction

Extracellular vesicles (EVs) are a heterogeneous population of membrane vesicles that are secreted from cells and act as key mediators of intercellular communication [1]. EVs were considered ‘cell dust’ until [2] the late 1980s, when Dr. Ross Johnstone discovered the formation of tiny membrane-bound vesicles during invagination of the reticulocyte membrane. EVs are known to enclose macromolecules such as lipids, protein, and genetic material, and also act as carriers of small non-coding microRNAs (miRNAs), which promote sequence-specific degradation of mRNA and function as inhibitors of translation [3]. EVs such as exosomes are formed during endosomal maturation while some are derived from plasma shedding in the case of microvesicles. They can exist in a wide range of sizes based on their biogenesis and composition [4,5]. Several published studies have used different terms to describe EVs based on their composition and sizes, and due to this diverse nature, a consensus classification has been used to separate EVs into three main subgroups, namely exosomes, microvesicles (MVs), and apoptotic bodies [6].

Bodily fluids such as saliva, urine, blood, semen, and breast milk are known to contain EVs, which are produced in response to various biological activities [4]. Notably, these vesicles are similar in size to many typical retroviruses, such as human immunodeficiency virus (HIV). This could potentially explain why studies on the EV–HIV relationship have revealed key similarities in the pathways involved in their intracellular formation [2,7]. Moreover, EVs such as exosomes, have been shown to play critical roles in disease pathogenesis for a number of viruses, including HIV [8], human adenovirus (HAdv) [9], and coronavirus (CoV) [10]. For instance, HIV is known to exploit the exosomal pathway to generate infectious particles that promote viral spread. A typical example is the dysregulation of cellular miRNA associated with modulation of viral infectivity and replication which HIV accomplishes by targeting and inhibiting the expression of specific host genes. HIV exploits transfer of vesicles and exchange of small regulatory non-coding miRNA from DCs to T cells via a ‘Trojan horse’ pathway to allow exchange of genetic materials between infected and non-infected cells, thus enhancing infection productivity [3,9,11,12,13,14]. Studies have further revealed that neural stem cell-derived EVs can significantly enhance cellular HAdv entry during infection via cellular surface ligands or receptors [9]. EV activity can therefore either exacerbate or inhibit viral diseases. However, the precise roles that EVs play in viral pathogenesis have not yet been fully elucidated, and therefore further study is warranted [15].

Immunological studies on EVs have further revealed their immunomodulatory potential. In particular, some of their activities include facilitating the intercellular communication between antigen-presenting and recipient cells, which induces the immune function of the latter [16]. EVs also package innate response signaling molecules that are specifically produced in response to viral infections. These cytokines possess antiviral properties that could render cells resistant in cases of reinfection [17]. Notably, among the many immunostimulatory roles that EVs play, this ability to induce secretion of antiviral cytokines, including interferons (IFNs), especially in upper and lower respiratory diseases, has intersected with recent studies on coronaviruses. As such, this growing research field is beginning to explore EV secretion as a relevant approach to diagnosing and potentially treating of CoV-related diseases [18,19]. Given the equivocal but crucial roles that EVs play in disease pathogenesis, spread, and mitigation for a number of viruses, this review intends to provide a broader perspective on some prominent virus–EV relationships and how they influence disease progression, with a particular focus on HIV and CoV.

## 2. Formation of Extracellular Vesicles (EVs)

EVs are known to be secreted by cells from all three domains of life—prokaryotes, archaea, and eukarya [20]. All EVs are enclosed by a lipid bilayer, some of which are formed as a result of the inward budding of the plasma membrane, these are known as exosomes, while some are formed via outward shedding resulting into microvesicular formation. This structure is relatively stable, thereby protecting vesicle cargo from enzymatic degradation [21,22]. They can exist in a number of different forms based on their biogenesis, namely exosomes, apoptotic bodies, MVs, oncosomes, ectosomes, etc. However, the most studied of these are the exosomes due to their dual importance for disease progression and inhibition [4,23]. Notably, the heterogeneity of EVs forms the basis for their classification and reflects how they are formed [21,24,25]. Before exocytosis, exosomes accumulate within a cell as endosome-derived vesicles, collectively known as multi-vesicular bodies (MVB). These are either targeted for degradation via a lysosomal pathway or are consistently released from living cells into the extracellular space. Some researchers have suggested that this process is a way to eliminate, alter, and/or transfer redundant cellular contents [22,23,26]. EVs, particularly exosomes, emanate during vesicular budding of the endosomal membrane, which gives rise to intracellular endosomal vesicles. Endosomes then fuse with the plasma membrane, and vesicles are released via exocytosis [11]. Exosomes range in size from 30–200 nm, whereas larger vesicles, like MVs, could span from 100–1000 nm [23,27]. Although both exosomes and MVs are secreted in response to typical physiological and pathological conditions, unlike exosomes that form within the lumen of MVBs, MVs are secreted via budding and shedding from the plasma membrane. Due to their heterogeneity and distinct cellular origins, a key challenge facing the study of EVs is how to effectively differentiate MVs from exosomes. MVs form a mixed population with exosomes during plasma membrane shedding, and this could be attributed to their near similar sizes, densities, and high-membrane lipid contents. Consequently, effective differentiation between them is only possible via gradient centrifugation and specific antibody immunoprecipitation [28,29]. EVs can be produced constitutively or induced due to a variety of pathophysiological processes, such as hypoxia, oxidative stress, senescence and cell death, inflammation/immune modulation, neurological diseases, and infections [22]. Importantly, they are usually released from certain cell types dependent on specific biological conditions, which suggests they may be suitable biomarkers for viral disease diagnosis.

### 2.1. Exosomes

Exosomes are typically the EVs with the smallest compartments, and as such, they share a size range with some viruses. These 30–200 nm vesicles are surrounded by a lipid bilayer that originates from the parent cell plasma membrane, and their release has been demonstrated in both in vitro and in vivo studies. Exosome development begins with the initial formation of an intraluminal vesicle (ILV) within an MVB, resulting from early endosomal maturation into the late endosome [10,22,30]. The MVB then fuses to the plasma membrane, and ILVs are subsequently released as exosomes [31]. Exosome distribution can be localized or systemic. Exosomes retain many endosome-related proteins after exocytosis, which they use for intracellular membrane fusion and transport to a recipient cell [32]. Proteins enriched in exosomal membranes include heat shock protein 70 (Hsp70), lysosomal protein (Lamb2b), fusion protein (CD9), and tetraspanin proteins, such as CD63 and CD81, which are known to interact with other transmembrane proteins, including α, β integrins, and peptidase. This composition distinguishes exosomes from other EVs [32,33]. Tetraspanin proteins, in particular, have been shown to influence EV formation and are crucial for exosome sorting, as they interact with miRNA and major histocompatibility complex (MHC) I and II immune recognition complexes. Tetraspanins also mediate fusion of secreted exosomes with target cells, and therefore exosomal tetraspanin (TSPAN) composition may impact target cell selection. A number of different TSPAN proteins have been associated with exosomes, including CD9, CD63, CD81, CD82, CD151, CD37, Tspan8, among others [34,35]. Two essential members of the Rab family, RAB27A, a key player in exosome size determination, and RAB27B are small GTPases that have also been shown to influence trafficking and docking of MVBs at the plasma membrane during the exosome formation [36]. After the release of exosomes into the extracellular matrix, they are taken up by recipient cells either through direct interaction or via receptor-ligand signaling [22,36]. The exosome-mediated transfer of secreted proteins and nucleic acids is known to be critical for many pathophysiological and biochemical cellular processes, as it allows the transfer of cellular content, independent of direct cell-to cell-contact. It also protects and preserves the 3D-structure of transferred molecules within the integrity of the vesicle lumen. Additional roles that exosomes play in cellular physiological processes include the elimination of unwanted proteins and redundant genetic materials, such as mRNA and miRNA [36,37,38], intercellular propagation of pathogens (e.g., viruses and bacteria), and facilitating immune stimulatory and inhibitory processes [10]. In organs such as the kidney, exosome secretion is crucial for the detoxification process [39]. Notably, the exosomal protein composition secreted by tumor cells differs from that of non-malignant cells [40]. For example, tumor cells are known to release vesicles containing protein antigens which they transfer to dendritic cells (DCs) in the cross-priming process for antigen presentation. Exosomes secreted during this process are termed ‘dexosomes’, and they have been shown to aid the priming of specific T cells and murine tumor cells in vitro [41].

### 2.2. Microvesicles

Unlike exosomes, MVs are cellular fragments formed through outward budding of the cellular plasma membrane and are released via exocytosis. These are 100–1000 nm in size and are made up of a pool of bioactive constituents. MVs were first described by Wolf in 1960, as vesicles derived from platelets [42,43]. Critically, they differ from exosomes in that they do not utilize the endosomal pathway by hijacking membrane constituents and cytoplasmic content for their formation and release [44]. EVs could be referred to as “exosomes” after the formation of vesicles (vesiculation) in the cytoplasm, but they are called MVs or microparticles (MPs) when vesiculation occurs as a result of lipid exchange between the inside and outside of the cell membrane [42]. Notably, MPs that are generated from the same cell may contain different lipid and protein content. This could lead to the repetition of such content intracellularly, thereby modulating cellular physiological homeostasis, and this can be detrimental when too many molecules are introduced. MV biogenesis requires an influx of calcium and cortical cytoskeleton, which enables the release of intracellular membrane-bound vesicles [45]. Viral-induced MVs are usually released from cells 24–30 h post-infection, and they may harbor viral content from infected cells either within or on their surfaces following outward blebbing [24]. Importantly, APCs, such as DCs, which act as cellular biosensors, can detect molecular signals necessary for immunity by acquiring MV enclosed-circulating antigens via pinocytosis, endocytosis, or phagocytosis [46,47,48,49].

## 3. Importance of EVs in Viral Infection and Pathogenesis

Vesicular interaction may occur between neighboring cells, immune cells, antigen-presenting cells (APCs) and recipient cells, cancer cells, and endothelial cells [1,4,23]. Studies on intercellular communication have revealed that vesicular budding from cells to produce the specific membrane-bound vesicles termed exosomes exhibits behavioral patterns that are targeted toward nucleic acid and protein packaging and transport. This makes them suitable biomaterials for drug delivery and nano-based pharmaceutical application [4,33], particularly, in the fast-growing fields of immune-therapy and development of targeted drug delivery systems [33]. Conversely, cargo loading, budding, shedding, and reacquisition of EVs by neighboring cells could support the spread of viral infection and promote disease progression. EVs have been shown to be involved in a number of immune-related activities, including autoimmune disease pathogenesis and the sequestering and transfer of innate cytokines and type I IFN response to viral antigens. Proviral effects of macrophage-derived exosomes include modulation of certain aspects of adaptive immune responses, some of which are antigen presentation and T cell function and polarization. In the case of HIV, EV-mediated transfer of the HIV co-receptors, CXCR5/CC5, to target cells that lack, or express low levels, of these receptors contributes to HIV infection by increasing the scope of susceptible target cells [50,51,52]. Exosomes can also facilitate drug resistance via lipid, miRNA, and protein transport, and similarly, it has been proposed that they can serve as a medium for metastatic signaling in cancer development by shuttling key proteins and miRNAs involved in oncogenic pathways. They could also mediate endothelial cell angiogenesis and facilitate the transformation of healthy cells into cancer cells [53]. Critically, the key roles the exosomes play in infectious disease pathogenesis further suggests that exosomes could be used as potential biomarkers for a number of such diseases, ranging from mild bacterial infection [54] to chronic viral diseases, with the potential for co-opting exosomes to prevent infection and/or spread. Most viruses share similar features with EVs, including their mode of entry, ability to mediate intracellular transfer of genetic materials (e.g., mRNA and proteins) [4], and packaging. MVBs within a cell are subjected to cargo loading via activity of the endosomal sorting complex required for transport (ESCRT), which is required for packaging, preferential sorting, and disposal of cellular contents [4,24]. Intriguingly, studies have shown that viral components associated with certain infections can be found in EVs, such as exosomes. This suggests that some viruses may co-opt MVB loading and EV formation pathways to promote their assembly and dispersal [23,24].

## 4. HIV

HIV has recently been classified as a chronic infection, with an aggressive incidence rate of over 0.5% as of 2018 and over one million new cases per year [15]. HIV is a positive-sense single-stranded RNA virus, belonging to the genus *Lentivirus* of the family Retroviridae [55]. It is the etiological agent of acquired immune deficiency syndrome, a viral disease that is characterized by progressive pathology and immune system depletion [56]. HIV specifically targets T-helper lymphocytes, DCs, and macrophages expressing the CD4 receptor for adherence and entry into cells. Despite the large number of studies on HIV and the continued focus on this disease, efforts to completely eradicate it are not presently feasible due to viral latency [15].

## 5. HIV and EVs

HIV utilizes a cellular pathway analogous to the formation of ILVs from MVBs, in which viral particles acquire their envelope structure from the membrane of infected cells and facilitate propagation through a mechanism similar to that of exosome release. To promote assembly, HIV hijacks the RAB27A pathway, which regulates the secretion of MVBs, as well as lysosomal organelles [4,54,57,58]. HIV-1 also exploits endosomal compartments and the ESCRT pathway, which enables activation of MVB genes and exosome release, to enhance the budding process [4,30,54]. The ESCRT pathway can be dependent or independent; however, both are crucial for HIV-1 budding and release. Specifically, the dependent pathway is required for HIV-1 budding via ESCRT component binding with the HIV-1 Gag protein, which enables budding off from the plasma membrane and eventual release into the extracellular milieu [59]. In neurodegenerative disease, HIV-Gag interacts with Aβ precursors and activates its cleavage, thus enhancing HIV protein synthesis (Table 1) [60,61,62,63]. Similarly, the independent pathway may facilitate viral content sorting in exosomes, which promotes viral release from infected cells. During these processes, tetraspanin proteins, ceramides, phospholipases, and other cellular components facilitate vesicular formation [34].

HIV-1 modulates vesicle secretion through its negative regulatory factor (Nef) protein, which is secreted in exosomes and modifies the intracellular trafficking pathways to enhance viral infectivity [20]. Notably, Mukhamedova et al. provided the first evidence that exosomes could act as biomarkers during HIV infection when they reported that exosomes isolated from Nef-expressing cells contain this protein [64]. Data further suggest that Nef released from HIV-infected cells in exosomes may promote systemic impairment of cholesterol metabolism in uninfected cells, which induces inflammation and contributes to multiple co-morbidities of HIV disease [64,65,66,67]. Intriguingly, Nef-expressing exosomes have also been implicated in development of neurodegenerative diseases, such as Alzheimer’s disease (AD). This likely occurs through a mechanism by which Nef released by HIV-infected microglial cells stimulates the release of inflammatory cytokines, which in turn promote the production and release of free radicals, leading to oxidative stress and excitotoxicity (Table 1) [60,61,68]. In addition, immune antiviral responses to HIV can be repressed by the Nef protein, thereby enhancing HIV’s ability to evade innate immune recognition in target cells [59].

Overall, the role of exosomes in HIV pathogenesis is complicated. Sims et al. reported that exosomes derived from breast milk, lung cells, and neuronal stem cells can mediate HIV-1 entry into T lymphoblastoid cells and macrophages in vitro. In addition, evidence suggests that HIV can exploit EV transfer from DCs to T cells as an alternative pathway for progressive infection, in a process termed transfection [64]. Serum EVs have further been shown to enhance infectivity of the human hepatitis G virus (which ordinarily has low morbidity and mortality) in patients co-infected with HIV [69], and another study revealed that short HIV-1 TAR element transcripts can be found in exosomes of HIV-infected cells [69]. However, HIV-1 entry and infection can also be inhibited by tetraspanin protein blockages, such as CD9 and CD81, and CD81-mediated inhibition of viral entry could indicate the specificity of exosomes in different cell types. Interaction between exosomes and HIV-1 is also partly mediated by viral envelope binding to T cell immunoglobulins and the mucin protein TIM4. These findings imply that exosome-mediated HIV-1 blockage may be a potential target for reducing HIV-1 infection [34,35].

## 6. HIV and the Trojan Exosome Hypothesis

An overview on retroviral biology, specifically focused on HIV, may help to better understand the increased susceptibility of HIV-specific activated memory T cells, as well as the elevated risk of infection observed for individuals vaccinated against HIV [81]. Retroviruses exploit pre-existing non-viral vesicle biogenesis pathways for the formation of infectious particles [82]. Thus, 16 years ago, it was proposed that HIV evades the host immune system by adopting patterns, structures, and composition analogous to that of endogenous exosomes [52]. Retroviruses, such as HIV, also co-opt the exosomal pathway to infect neighboring cells via receptor- and envelope-independent uptake. Accordingly, this pathway has been implicated in HIV transmission, while simultaneously offering insight into potential strategies for inhibiting disease development, through a model termed the ‘Trojan exosome’ hypothesis [52,81,83]. This model incorporates the relationship between the complex HIV release pathway, its acquisition of host phenotype, and its distinctive lipid composition [81]. Retroviruses use exosome conduit for intercellular transfer and entry in an envelope-independent manner to facilitate disease progression [84]. In the context of the Trojan exosome hypothesis, retroviruses and exosomes usually carry similar but increased lipid contents (e.g., glycosphingolipids and cholesterol) relative to the parent cell membrane [58]. Comparative analysis has further revealed that they also contain similar protein content profiles, including those derived from the plasma membrane (e.g., tetraspanin, integrin, MHCs, and Lamp) and cytoplasmic proteins (hsp, actins, etc.) [57,58]. Similar to lipids, these proteins are present not just in trace amounts, but could be more abundant than some of their endogenous counterparts [65]. In an infected cell, HIV uses its Gag protein to target and bind ILV components, such as TSG101 and cyclophilin, thereby facilitating the formation of larger aggregated particles.

**Figure 1:**
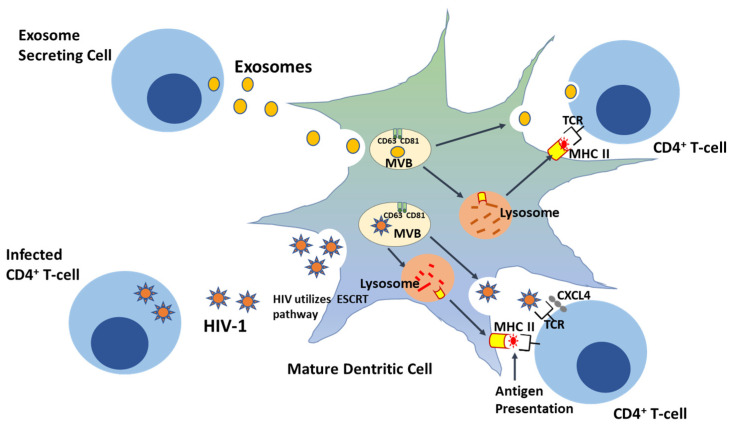
HIV-1 exploits the ‘Trojan exosome’ transdissemination pathway—a pre-existing exosomal sorting route—during transinfection in CD4+ cells.

One of the essential tenets of the Trojan exosome hypothesis is that the infectivity of retroviruses is low, independent of their *Env* and surface proteins [64,83]. For example, analysis of the *gypsy* and *mdg3* retroviruses both in vitro and in vivo found no significant difference between Env-independent infection and infection in the presence of the *Env* gene. Retroviruses are relatively resistant to the adaptive immune response [85,86], and HIV, in particular, has been shown to preferentially infect activated memory CD4+ T cells [81,83]. Notably, the ability to infect CD4+ cells was found to be impeded in an HIV mutant lacking the *Env* gene, indicating a crucial role for Env in facilitating viral attachment and entry into these cells [87]. However, no substantial difference in infectivity rate was observed between *Env*^−/−^-HIV and the wild type viruses, which suggests that HIV entry depends on more than just one pathway [86]. HIV-1 uptake by APCs, such as mature DCs, could follow the ESCRT exosome sorting pathway, an inherent DC pathway for antigen processing and presentation, allowing transinfection of HIV-1-CD4+ T cells (Figure 1). Hijacking this exosomal sorting pathway enables viral particles to evade host immune responses and could indirectly activate APCs, such as DCs, for functional epitope MHC II complex antigen presentation to CD4+ T cells [88]. The Trojan exosome hypothesis thus predicts that an effective antiretroviral immune response must inactivate retroviruses in infected cells without previous exposure or prior to the first round of retroviral replication in host cells [81]. It also predicts that antiretroviral immune responses must be targeted against highly polymorphic host cell exosomal antigens, and finally, the detection of non-self, polymorphic, host cell exosome antigens should stimulate a variety of effective naïve responses targeted at eliminating foreign antigen [83]. The Trojan hypothesis thus suggests a retroviral system of development and transmission as a function of their physical and functional properties. Although the aggressive HIV mortality rate, which was at its peak between 2003 and 2004, with approximately 20 million deaths and approximately 15,000 deaths daily has since declined over the years, with less than 700,000 deaths globally in 2019, therapeutic approaches have since been developed in order to sequester viral replication in infected individuals [89].

## 7. Coronavirus

Coronaviruses encompass a large group of pleomorphic [19] zoonotic enveloped viruses, containing large non-segmented, single-stranded, positive-sense RNA genomes. They have previously been identified as the causal agents of severe acute respiratory syndrome (SARS) and Middle East respiratory syndrome (MERS) [90]. Recently, a novel coronavirus termed SARS-CoV-2 that causes a severe acute respiratory condition known as coronavirus disease 2019 (COVID-19) has been reported to have emerged in Wuhan city of Hubei province of China in late 2019. This infection was subsequently classified as a Public Health Emergency of Global Concern by the World Health Organization in January 2020. SARS-CoV-2 belongs to a distinct clade of coronaviruses designated as beta-CoV, a group that includes both MERS-CoV and SARS-CoV, and it is believed that the divergence of SARS-CoV-2 from SARS-CoV is sufficient for it to be considered a unique human infecting strain [91,92]. Critically, there is currently no known effective prophylaxis or precise antiviral treatment against SARS-CoV-2 infection [19].

As with other viruses, the mechanisms of entry, replication, exit, and dissemination of coronaviruses may be influenced by the production of EVs in infected cells [56]. In particular, several viruses enter exosomes or extracellular double-membrane vesicles (DMVs) after initial infection, following viral synthesis, and during cell-to-cell spread. In the case of SARS-CoV, studies have shown that infection of AT2 cells with this virus led to the migration of viral particles within DMVs in vitro, and synthesis of SARS-CoV viral RNA is initialized in virus-induced membrane vesicles. Virion budding and release begins inside the cell cytoplasm, after which it progresses into the endoplasmic reticulum and the intermediate compartment, prior to release of newly formed viruses as secretory vesicles via exocytosis [93]. A post-mortem analysis of human renal samples further confirmed that SARS-CoV-2 can be found within DMVs, as demonstrated by histopathological visualization of immunostained samples via light and electron microscopy. SARS-CoV-2 was also found to be associated with vesicle packaging based on the observation of crown-like viral particles encapsulated by electron-dense spikes adjacent to a DMV. This suggests the possibility of viral particle assembly and exosomal cellular transport [93,94]. Notably, recent studies have proposed an improved competitive inhibition therapy that uses EVs with an affinity for the virus to inhibit SARS-CoV-2 binding to angiotensin-converting enzymes positive (ACE2+) type 2 alveolar cells. This therapy thus increases the chance of preventing downregulation and inhibition of ACE2 activity. In addition, small EVs have been used to alleviate hyper inflammation in severe SARS-CoV-2 patients [90,91,95,96,97]. It has further been shown that SARS-CoV-2-infected mesenchymal cells produce ACE2+ EVs. These EVs contain signals that activate the phagocytic activities of cells such as monocyte and/or neutrophils, inducing anti-inflammatory effects and leading to reduced pathology in the lungs [10,95,98,99]. Vikram Sengupta et al. also reported that allogeneic bone marrow mesenchymal cell (MSC)-derived EVs, particularly exosomes termed ExoFlo, are an efficacious treatment for severe SARS-CoV-2. Notably, these have shown promising results when administered to patients, reducing the use of invasive oxygen support and mechanical ventilation, and resulting in an 83% survival rate and improved oxygenation in the upper and lower respiratory tract. However, additional random clinical trials will be needed to validate these findings [100,101].

## 8. Immunological Function of EVs

**Figure 2:**
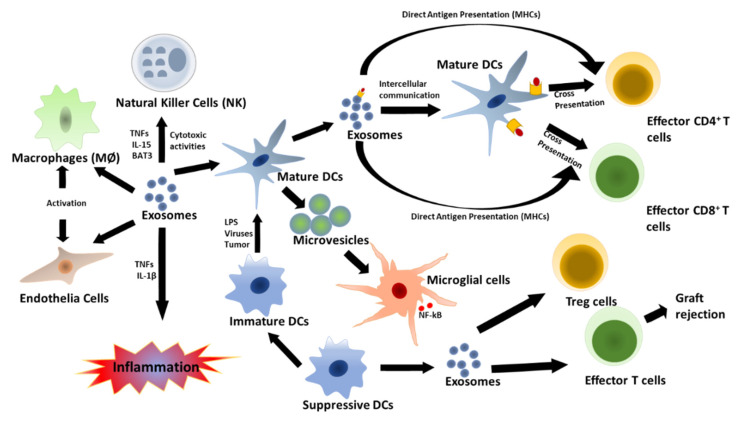
Innate and acquired immune responses regulated by DC-derived exosomes. Mature DCs can use exosomes for antigen presentation to T cells. This induces an innate immune response and proinflammatory cytokine secretion via host factor-mediated immune activation. Mature DC-derived MVs can then stimulate cytokine secretion by microglial cells. However, exosomes secreted by immature DCs can become immune suppressive, promote activation of T-reg cells, and induce apoptosis of effector T cells.

The innate immune response specifically employs a variety of immune cells that may be involved in inflammation or phagocytosis, dependent on the specific signaling pathway, and evidence has shown that these innate immune cell-signaling compounds can be carried by secreted EVs [100,101,102]. Thus, it is believed that EVs may play a crucial role in the development and sequelae of immunity during both adaptive and innate immune responses, as evidenced by the presence of MHC class I and II molecule on EVs (Figure 2). However, the precise effects of protein enriched EVs on immune cell function have yet to be fully elucidated. Upon infection in humans, pathogens are detected by specific receptors, including pattern recognition receptors (PRRs), Nod-like receptors (NLRs), and Toll-like receptors (TLRs), which recognize pathogen-associated molecular patterns (PAMPs) and activate host immune responses. When these immunological receptors detect viral dsRNA, they activate expression and secretion of proinflammatory cytokines, type I IFN, interferon regulatory factor (IRF)3 and IRF7, and nuclear factor (NF)-κB [102,103].

One of the best-studied viral infections is HIV-1. This enveloped retrovirus uses both primary and secondary receptors and viral ligand signaling for host cell entry. In particular, HIV-1 uses the CD4 host cell surface receptor for the initiation of binding and cell entry. Co-receptors further include the CCR5 and CXCR4 chemokine receptors. In addition, HIV-1 has been found to exploit other non-conventional modes of infection, distinct from CD4-mediated entry, which involve recruitment of unique molecular signatures for entry [27,35,36,38,103]. Critically, EVs have been shown to contribute to immune response evasion by HIV-1 particles via the masking of antigens and viral PAMPs [2]. Conversely, CD4+ T cell-secreted exosomes can prevent HIV infectivity via binding of the viral envelope protein gp-120, thus preventing it from binding to the CD4 receptor on target cells. CD4+ exosomes also help transport antiviral effector molecules intercellularly, leading to inhibition of viral propagation [52], and virus-infected cells may secrete EVs carrying dsDNA, which induces an innate immune response [100].

Exosomes secreted by DCs have also been associated with innate immune response activation. Both immature and mature DC-derived exosomes can carry tumor necrosis factor (TNF) superfamily signaling cytokines, such as FAS ligand and TNF-related apoptosis-inducing ligand (TRAIL) on their surfaces, and they activate natural killer (NK) cells by interacting with NK cell surface receptors [27,31,36,38]. In addition, exosomes play key roles in both direct and indirect antigen presentation by DCs. Direct antigen presentation involves sequestration of circulating exosomes by DCs (Figure 1). In this process, extracellular exosomes are taken up by mature DCs and used to present antigenic peptide-MHC II complex to activate effector T cells; this is known as antigen cross presentation [27,103]. On the other hand, mature DCs can take up circulating exosomes carrying antigen or peptide and present them on their endogenous MHC molecules for specific T cell activation. Lastly, DC-derived MVs may also promote immune activation by inducing NF-κB activation in microglial cells, and this has been found to be an inflammatory response marker in central nervous system (CNS) autoimmune encephalomyelitis [31,36,88,104].

## 9. Autophagy Pathway

The development of naked virus (especially enteroviruses)-containing EVs is not yet well understood. These EVs could be virus specific, which might involve secretory autophagy, a degradative pathway employed by most eukaryotes [20,105]. Autophagy is a phenomenon that occurs as a result of pathological and stressed physiological conditions and leads to self-engulfing, breakdown, and recycling of damaged cytoplasmic content. In this process, autophagosome-enclosed damaged cytoplasmic material fuses with the lysosome, and its contents are degraded by lysosome hydrolase. In the case of viruses, however, the enclosed virion fuses with the cellular plasma membrane to eliminate its viral content [20,105,106]. Autophagy can be induced by the p53 tumor suppressor protein following the cellular stress response, and it may also be accompanied by apoptosis, senescence, or cell cycle arrest [105,106]. Another regulatory protein, miR-210, also regulates autophagy by targeting inhibition of Atg7, leading to the differentiation of lung fibroblasts to myofibroblasts [107]. Notably, a role for EV-associated autophagy has further been identified in numerous conditions. For example, studies have shown that in breast cancer cells, EV-induced autophagy can influence the tumor microenvironment, promote tumorigenesis, and induce both reactive oxygen species (ROS) and DNA repair [108]. In addition, during pregnancy in humans, miRNA-containing EVs derived from the trophoblast cells that form the outer layer of the blastocyst and eventually give rise to the placenta have been shown to inhibit viral replication by inducing autophagy in an infected cell, thereby protecting the fetus from viral infection. This viral replication inhibitory mechanism has also been observed in HIV-1, rubella, and varicella infection [38]. To combat this, some viruses have acquired and developed ways to manipulate autophagy so as to promote their transmission. In particular, HIV-1 and herpes simplex virus inhibit autophagy complex maturation by interacting with Beclin-1, a regulator of autophagy and apoptosis [20].

## 10. EVs as Diagnostic Agents

Exosomes secreted in bodily fluids, including urine, saliva, and blood, are known to contain lipids, proteins, and miRNAs, making them a suitable target for disease diagnosis. Minimally or non-invasive exosome-based diagnostics using bodily fluids would provide less-painful, stress-free, and low-cost alternatives to conventional techniques [109], and there is an increasing body of evidence to support this strategy. For example, transcriptomic analyses of serum miRNAs in lung and colorectal cancer patients revealed a pattern consistent with the respective diseases [109,110], and serum levels of miR-141 were able to distinguish prostate cancer patients and healthy individual. In addition, compelling evidence showing similarities between miRNAs in circulating exosomes and those in cancer cells further supports their utility as a suitable diagnostic tool [111,112]. This also extends beyond cancer, as a similar analysis of serum miRNA in pregnant and non-pregnant women found that an elevated level of human placenta miRNAs correlated with a particular phase of pregnancy [111]. In another study, CD4 polymorphisms were identified as an indicator of disease risk and progression for HIV, multiple sclerosis, chronic hepatitis B, and giant cell arteritis when amniotic fluid-derived exosomes were obtained for diagnostic screening [42].Thus, exosome content could serve multiple diagnostic functions, by elucidating physiological states or disease risk, acting as a representative of actively secreting cancerous or infected cells, or revealing metastatic signaling in distal cells [109,113].

Accordingly, exosomes secreted in bodily fluids have been proven to be suitable biomarkers for HIV diagnosis. Exosomes isolated from semen target multiple phases of the HIV-1 lifecycle, and therefore, these may regulate the immune response to infection in order to restrict viral replication. The presence of miRNAs and other biomarkers, such as CD59, an inhibitor of the complement system, as well as CD46 and CD59, in exosomes isolated from bodily fluids of HIV patients has further stimulated a large number of studies aimed at investigating the diagnostic and prognostic potential of EVs [59]. In one antiretroviral therapy study, examination of plasma EVs from HIV-1 patients showed an increased abundance of exosomes and exosome-associated protein, such as TSG101, in infected patients as compared to uninfected subjects, and this result was also correlated with CD4/CD8 ratio. The presence of acetylcholinesterase in EVs was further found to correlate with the length of infection, independent of treatment status, disease progression, and patient age. These findings support the use of EVs as biomarkers for HIV infection and progression [103,114].

Saliva and urine exosomes have proven to be reliable indicators in prostate cancer and urogenital disease. However, their cargo protein and nucleic acid content may also be rich enough to serve as potential biomarkers for causative organism screening in saliva and urine-based diagnostics [12,109,115]. For example, saliva, among other bodily fluids, has been found to be a key tool in the diagnosis of SARS-CoV-2 infection. According to Meng Xu et al. 2020, the United States Food and Drug Administration approved the first emergency saliva testing for COVID-19 diagnosis on 14 April 2020. This breakthrough diagnostic tool was developed at the Human Genetics Institute of New Jersey, Rutgers University, New Jersey and was the result of a collaboration between Rutgers’ RUCDR Infinite Biologics, Accurate Diagnostic Labs, and Spectrum Solutions [116]. To date, immunological and molecular screenings such as enzyme-linked immunosorbent assay (ELISA), reverse transcription (RT)-PCR, RT-loop-mediated isothermal amplification (LAMP), and lateral flow immunoassay (LFIA) have all been used to detect viral transcript and viral load in saliva samples [116,117].

Czumbel et al. 2020 proposed various ways in which SARS-CoV-2 could make its way into saliva, among which are via nasopharyngeal epithelium draining into the mouth, infected oral mucosal endothelial cells, secretion from infected salivary glands, blood plasma-crevicular fluid route to the oral cavity, and periodontal tissue exudate [115,117,118,119,120]. The basis for the Rutgers saliva test can be traced to the ability of exosomes to transfer the ACE2 receptor, a human angiotensin-converting enzyme 2 receptor to recipient cells following fusion and internalization. This process takes place through ACE2 interaction with the S protein of SARS-CoV-2 prior to entrance into the cell, suggesting a role for exosomes in viral pathogenesis [116]. Exosome cargo, such as tetraspanins, may also influence SAR-CoV entry. Studies have also revealed that CD9-enriched exosomes enhance viral uptake by recipient cells and promote susceptibility to infection [19]. Exosomes secreted by ACE2-overexpressing endothelial progenitor cells have further been shown to enhance the function of microvascular endothelial cells and to confer protection against injury, suggesting the importance of exosomal ACE2 in disease pathogenesis [121].

## 11. EVs as Therapeutic and Drug Delivery Agents

EVs are fast emerging as a promising alternative to cell-based therapy, with numerous advantages, such as a long shelf-life, the possibility of lower clinical damage that may develop due to an unfavorable disease microenvironment, and lower tumorigenic impact [122]. Based on the increased recognition of the myriad roles that EVs play in a growing number of infectious diseases, their diagnostic and therapeutic application is expected to surge in the coming years [28]. Notably, the exponentially growing field of exosome biology now encompasses both their functions in the body, as well as more practical applications in disease diagnostics and therapeutics [109]. For example, studies on exosome-assisted HIV-1 uptake have shown that human-derived exosomes aid virus entry into cells, but this mechanism of exosome-mediated entry can be blocked by CD81 and CD9 antibodies. The sequestering of HIV-1 entry could thus represent a promising therapeutic approach to treating latent HIV infections, especially in immune-privileged sites like the brain [35].

Importantly, clinical data support the use of exosome-based therapeutics. Bernard Escudier et al. 2005 performed the first Phase I clinical trial using autologous DC-derived exosomes pulsed with melanoma-associated antigen (MAGE) 3 peptides to promote immune response in stage III/IV melanoma patients. This process involved the use of DC-derived exosomes complexed with MHC class I loaded with peptide to promote tumor rejection and bolster T cell immune responses [109]. The resulting data show that carefully generated exosomes have a promising impact on tumor suppression. In another Phase 1 clinical study in 2005, the efficacy, toxicity, safety, and feasibility of DC-derived exosomes loaded with tumor antigens was assessed in patients with non-small-cell lung cancer (NSCLC). Here, DC-derived exosome vaccines were found to be feasible and well tolerated at various concentrations in patients with advanced NSCLC. Long-term stability and activation of NK cells and other immune effectors further suggest that DC-derived exosomes are a promising therapy for NSCLC patients [41].

In addition, many published studies support the use of EVs for the development of effective EV-based drug delivery strategies. EVs possess a number of advantages over many traditional drug delivery mechanisms and therapies, including the fact that they contain a naturally occurring cargo of biomolecules. Studies have shown, for example, that an EV-based drug delivery system for suppression of HIV-1 reservoirs in the spinal cord and the brain was able to reduce the symptoms of HIV-associated neurocognitive disorder. Recently, cutting-edge studies have demonstrated the potential use of EVs as a drug delivery system for encapsulating small molecules, such as mRNA, RNA, peptides, proteins, and a host of other biomolecules, for the treatment of various viral diseases [13,33,98,99]. A novel therapeutic application of exosomes in the central nervous system was demonstrated by Zhuang et al. in a trial that involved the treatment of neuroinflammatory disease using a non-invasive exosome-based delivery technique. Specifically, they performed exosome-mediated delivery of signal transducers and curcumins specifically aimed at alleviating pathological responses in microglia cells, thereby demonstrating a unique, non-invasive method of drug delivery [123]. Exosome therapies are somewhat analogous to cell immunotherapy in their approach and application, and both involve the use of naturally occurring biological products. However, despite these similarities, exosomes are easier to manipulate since they are metabolically inactive molecules. The only known constraint to exosome-mediated therapy compared to cellular immunotherapy is that there are no defined immunological allotypes for treatment reference [98,109]. Because exosomes used for therapy are obtained from the same patients they are used on, further study on de novo synthetic exosomes and compatibility is critically needed [109].

## 12. Discussion

EVs are secreted by cells in virtually all tissues, organs, and bodily fluids, which is reflected by their abundance in the body, and their secretion can be either systemic (blood) or localized. EVs also play critical roles in viral disease pathogenesis and progression, with activities that can either lead to the exacerbation or improvement of disease, depending on the signal they carry. For instance, in regards to HIV and Epstein–Barr virus infection, it has been shown that EVs carry viral proteins and or nucleic acids that aid their reactivation during latency, this in return impedes host immune response to virus [100]. It was also reported that hepatitis C virus (HCV) protein and RNA were found in exosomes purified from plasma of patients with HCV—viral RNAs were packaged within exosomes that were secreted from infected hepatocytes [124]. A similar study also showed that HCV secreted EVs from infected patients facilitated intercellular viral propagation by transferring a complex of HCV replication element RNA and proviral loading molecule Ago2-miR122-HSP90 via an interaction with 5′UTR of viral RNA [19]. Herpesviruses such as Herpes Simplex Virus type-1 and Human cytomegalovirus exploit the EV biogenesis pathway as a means of exit and their maturation also depends on ESCRT machinery while bearing EV-associated cellular makers [19,20,125]. Their importance is further evident in the development of exosome-based disease diagnostics, therapeutics, and targeted drug delivery systems. In particular, evidence suggests that EVs can act as disease biomarkers and facilitate therapeutic development for viral diseases, such as HIV-1, SARS-CoV-2, and HAdv. An exosome-based vaccine study, reported by Anticoli in 2018, revealed that an exosome-derived vaccine candidate (consisting of a DNA vector expressing the E7 protein of human papilloma virus fused with the C-terminus of exosomes carrying the Nef protein) was able to stimulate cytotoxic T lymphocyte response against the E7 protein of human papilloma virus [126]. EVs may induce activation of regulatory molecules, which could inhibit the replication of HIV-1 and other viral diseases. The use of EVs in clinical diagnostic and therapeutic applications has also replaced some traditional invasive and semi-invasive techniques, such as incisions and needle sticking, thereby reducing the pain and discomfort associated with such practices. Therefore, EV analysis and profiling may hold the key to personalized medicine, as well as infectious disease mitigation and/or control.

## Figures and Tables

**Table 1:** Extracellular vesicle-borne HIV markers implicated in neurodegeneration.

HIV-Associated Markers Found in EVs	Site and Mechanism of Action	Consequences of Action	Results and Reference(s)
Tat	HIV-infected microglia, macrophages, and CNS cells, induce inflammation and secrete viral proteins, such as Tat and gp-120	Inflammatory stimulus leads to a cycle of excessive cytokine and chemokine production (e.g., Aβ and ROS); Tat-expressing astrocytes and HIV-infected cells are taken up by neurons, leading to neuronal injury and death	Neuroinflammation, excitotoxicity, induction of oxidative stress, and blood–brain barrier damage [62,70,71]
gp-120	HIV-infected microglia, macrophages, astrocytes	Infected neurons release proinflammatory cytokines that stimulate activation of N-methyl- D-aspartate receptors; when secreted in excess, this promotes excitotoxicity and production of free radicals, such as ROS	Excitotoxicity and oxidative stress [60,61,68]
Gag	Gag binds the Aβ precursor, APP, which sequesters this protein in lipid rafts within macrophages to inhibit viral replication and spreading; Gag then promotes cleavage of Aβ	Decreases Aβ production and accumulation	HIV protein synthesis [60,61,62,63,68,70]
ALIX, CD9, CD63, and CD81	Localized within amyloid plaques in the brains and post-mortem tissues of human AD patients	Increases spread of the pathogenic AD proteins	Oxidative stress and neuropathology [72,73,74]
Flotillins	Found within amyloid plaques in brains from an AD mouse model and in post-mortem tissues of human AD patients		HIV-associated neuropathology [72]
Tau	Aggregates as hyperphosphorylated *tau* in neurofibrillary tangles	Neuron-to-neuron transportation of *tau* contributes to the pathogenesis of AD	Associated with neurodegeneration and neuropathological changes [72,75,76,77]
APP	Associated with synapse organization, synaptic signaling, cognition, and neurogenesis	Cleavage and endocytic transportation of APP are critical for packaging Aβ into exosomes for dispersion	Neurodegeneration, neurodegenerative disorders, HAND, and neuropathological changes [72,78]
Aβ	Found within the brain tissue of those with AD and in HIV patients; accumulation of Aβ in the brain occurs with aging and is an important pathological event in AD	Damages the BBB, and could potentiate the development of AD-like pathology in the HIV infection	Aβ participates in AD pathophysiology and elevated levels have been reported in the brains of patients with HIV and Tat-exposed neuronal cells [72,79]
CD30	Produced in CSF in high concentration	Higher concentrations in CSF correlates with higher concentrations of NFL	HAND, autoimmune encephalitis [75]
Nef	Functional Nef is delivered to HIV virions and Nef- containing exosomes fuse with bystander cells and induce apoptosis	Nef induces dramatic dysregulation of cellular and exosomal miRNAs in human monocytic cells	Excitotoxicity and oxidative stress [60,68]
Vpr	Activates the NLRP3 inflammasome in human microglia	Induces HIF-1α transcription; oxidative stress-mediated neurotoxicity in HIV patients results from direct neuronal injury by HIV viral proteins	Neurodegeneration and neuropathological changes [8]
L1CAM (cell adhesion molecule)	L1CAM+ neuronal-derived EVs found in the brain neuron and serum of HIV-1 patient	L1CAM+ EVs induces neuroinflammation and cause brain damage	Neurodegeneration, cognitive impairment [77,78,80]
NCAM	Associated with synapse organization, synaptic signaling, cognition, and neurogenesis	Induces neuroinflammation and brain injury	Neurodegenerative disorders [73,78,80]
HMGB1	Localized in the brains of subjects with AD, and colocalized with Aβ in senile plaques	HMGB1 is actively secreted by necrotic or injured cells initiated by immune cells in the brain; results in brain injury and neuroinflammation when secreted into the extracellular space	Neuroinflammation, traumatic brain injury, neuronal damage and cognitive impairment [73,77,80]
TIM-4	Isolated from CSF exosomes, plasma, and CSF	Spread of α-synuclein between neurons via the exosome route confers cytotoxicity to recipient cells	Neurodegeneration and neuropathological changes [72]
TNF-α, IL-1-β, and IFN-γ	Produced by infected monocytes and T cells, as well as activated microglia and astrocytes	Produces MIP-1, causes inflow of immune cells into the CNS, and contributes to neuroinflammation and injury	Neuroinflammation and brain injury [80]
CD14	Produced in plasma and CSF	Excess soluble CD14 facilitates severity of cognitive impairments and risk of death	Cognitive and neurodisorders [74,80]

Abbreviations: Aβ, amyloid-beta; AD, Alzheimer’s disease; APP, amyloid precursor protein; BBB, blood–brain barrier; CNS, central nervous system; CSF, cerebrospinal fluid; HAND, HIV-associated neurocognitive disorder; IL, interleukin; MIP-1, macrophage inflammatory protein 1; NEF, negative regulatory factor; NFL, neurofilament light; ROS, reactive oxygen species; TNF, tumor necrosis factor.
